# Antibodies against Two Testudinid Herpesviruses in Pet Tortoises in Europe

**DOI:** 10.3390/ani12172298

**Published:** 2022-09-05

**Authors:** Christoph Leineweber, Rachel E. Marschang

**Affiliations:** Laboklin GmbH & Co. KG, Steubenstr. 4, 97688 Bad Kissingen, Germany

**Keywords:** tortoise, *Testudo*, testudinid herpesvirus, virus neutralization, serology

## Abstract

**Simple Summary:**

Herpesviruses are important pathogens in tortoises and cause latent infections. Serological testing is therefore an important tool for the detection of herpesvirus-infected tortoises. This retrospective study describes the detection of antibodies against two herpesviruses in pet tortoises in Europe. Of the 1728 samples tested, antibodies against one or both of the viruses used were detected in 122 (7.06%) of the tortoises. Detection rates differed depending on virus type, host species, and year of sampling. For individual viruses, detection rates also differed depending on season and country of origin. A better understanding of both the herpesviruses’ prevalences and the immune response to infection will help protect these animals in future.

**Abstract:**

Herpesviruses are important pathogens of tortoises, and several serologically and genetically distinct virus types have been described in these animals. Virus neutralization testing is commonly used in Europe to determine previous infection with the two types most often found in pet European tortoises, testudinid herpesvirus (TeHV) 1 and 3. In this retrospective study, the results of serological testing for antibodies against each of these viruses in serum or plasma samples from 1728 tortoises were evaluated, and antibody detection rates were compared based on virus type, host species, year, season, and country of origin. Antibodies (titer 2 or higher) against at least one of the two viruses used were detected in a total of 122 (7.06%; 95% CI 5.95–8.37%) of the animals tested. The antibody detection rates differed significantly depending on the tortoise species (*p* < 0.0001) and the year of sampling (TeHV1 *p* = 0.0402; TeHV3 *p* = 0.0482) for both virus types. For TeHV1, antibody detection rates differed significantly (*p* = 0.0384) by season. The highest detection rate was in summer (5.59%; 95% CI 4.10–7.58%), and the lowest was in fall (1.25%; CI 0.53–2.87%). TeHV1 antibody detection rates did not differ significantly (*p* = 0.7805) by country, whereas TeHV3 antibody detection rates did (*p* = 0.0090). The highest detection rate, 12.94% (95% CI 7.38–21.70%), was found in samples from Italy. These results support previous hypotheses on the species’ susceptibility to TeHV1 and 3 and the use of serology as a diagnostic test for the detection of herpesvirus-infected tortoises.

## 1. Introduction

Herpesviruses have often been described in tortoises and can be associated with severe disease, most often of the upper digestive and upper respiratory tract [1]. These viruses are considered a significant threat to the health of pet tortoises and can cause significant disease outbreaks with high morbidity and mortality rates [1]. So far, four different herpesviruses have been described in tortoises. Testudinid herpesvirus 1 (TeHV1) was first described in association with a disease outbreak among Russian (*Testudo* [*Agrionemys*] *horsfieldii*) and pancake tortoises (*Malacochersus tornieri*) in Japan [2]. It has also been described in pet tortoises in Europe repeatedly [3,4,5]. Testudinid herpesvirus 2 (TeHV2) has been described in Agassiz’s desert tortoises (*Gopherus agassizii*) in the USA [6] and has also been reported in a single case in a Texas tortoise (*Gopherus berlandieri*) kept in Spain [4]. Testudinid herpesvirus 3 (TeHV3) is in the species *Testudinid alphaherpesvirus 3*, the only tortoise herpesvirus species classified by the international committee on taxonomy of viruses (ICTV). TeHV3 has been found in a wide range of species in Europe and in other parts of the world [1,4]. Testudinid herpesvirus 4 (TeHV4) has so far only been detected in African species in the USA and in Europe [4,7,8]. All of these viruses have been shown to cluster within the subfamily *Alphaherpesvirinae* together with other chelonian herpesviruses in the genus *Scutavirus* [7,9].

Since herpesviruses cause latent infections, detection of clinically inapparent infected animals that may not be shedding virus is an important diagnostic tool. Serological testing has been described for the detection of antibodies against TeHV1, 2, and 3. The virus for which the most data on serological testing have been published is TeHV3. This virus has been used in neutralization tests and in enzyme-linked immunosorbent assays (ELISAs) for the detection of antibodies. TeHV3 isolated from a Hermann’s tortoise (*Testudo hermanni*) in terrapene heart cells (TH-1) has been used in a number of studies for the detection of antibodies in several tortoise species [10,11,12]. An ELISA has also been described for the detection of antibodies against TeHV3 in Mediterranean tortoises [13]. A study comparing herpesviruses isolated from tortoises showed that the two types included in the study (later shown to be TeHV3 and a single TeHV1 isolate) did not cross react serologically [14], demonstrating the necessity of using the virus type of interest for serological testing. In contrast, a study using an ELISA with a TeHV3-specific antigen for the detection of antibodies in wild Agassiz’s desert tortoises revealed a high (30.9%) antibody prevalence [15]. The authors hypothesized that this could be due to cross-reactivity between TeHV3 and TeHV2, since only TeHV2 was found in tortoises from the same population in virus detection studies. So far, only TeHV1 and TeHV3 have been isolated in cell cultures and are available for serological testing. These are also the two virus types most often found in pet tortoises in Europe [3,4].

The aim of this retrospective study was to provide an overview of serological testing for antibodies against TeHV1 and TeHV3 in pet tortoises in Europe. It was hypothesized that antibody detection rates would differ significantly depending on the host species and virus type and that these rates would correlate to polymerase chain reaction (PCR)-based virus detection rates previously reported in pet tortoises in Europe according to host species, virus type, country of origin, and season.

## 2. Materials and Methods

Plasma and serum samples submitted to a commercial veterinary laboratory (Laboklin GmbH & Co. KG, Bad Kissingen, Germany) between January 2016 and December 2020 were evaluated in this retrospective study. Samples were submitted for diagnostic testing by veterinarians and owners. Reasons for testing were not provided. All samples were heat treated at 56 °C for 30 min for compliment inactivation and stored at 4 °C for up to 5 days before testing. Neutralizing antibodies against a TeHV1 isolate from a Russian tortoise (1301/B99R/97, GenBank DQ343883.1) and a TeHV3 isolate from a Hermann’s tortoise (4295, [16]) in TH-1 were detected as described previously [10,11], except that plates for both antibodies were read after 7 days of incubation at 28 °C in order to lower turn-around time. Plasma from Hermann’s tortoises that had previously tested negative for both TeHV1 and TeHV3 antibodies were used as negative controls; plasma from Russian tortoises that had previously tested positive for antibodies against TeHV1 but not TeHV3 was diluted to a titer of 8 and used as a positive control for TeHV1 antibody testing; and plasma from spur-thighed tortoises (*Testudo graeca*) that had previously tested positive for a antibodies against TeHV3 but not TeHV1 was diluted to a titer of 8 and used as a positive control for TeHV3 antibody testing. Titers between 2 and 4 were considered suspect positive, titers of 8 low positive and titers of 16 and greater were considered positive. Plasma or serum dilutions associated with cytotoxicity were not evaluated, and only higher dilutions in which cytotoxicity was no longer observed were included. If no neutralization was detected in these higher dilutions, the sample was considered negative. Tests in which virus titration controls or positive or negative plasma controls did not provide the expected results were not evaluated, and samples were retested.

The statistical analyses were carried out using the statistical analysis software (SAS) (SAS Institute, Cary, NC, USA) for the calculation of the positivity rates. The 95% binomial confidence intervals were calculated based on the Wilson procedure [17]. Binary logistic regression was used with cut-off for significance of *p* ≤ 0.05 for comparisons of the positivity rates between the factors species, year, season, and country of sample origin. The seasons were divided as follows: March to May were classified as spring, June to August as summer, September to November as fall, and December to February as winter samples.

## 3. Results

**Figure 1:**
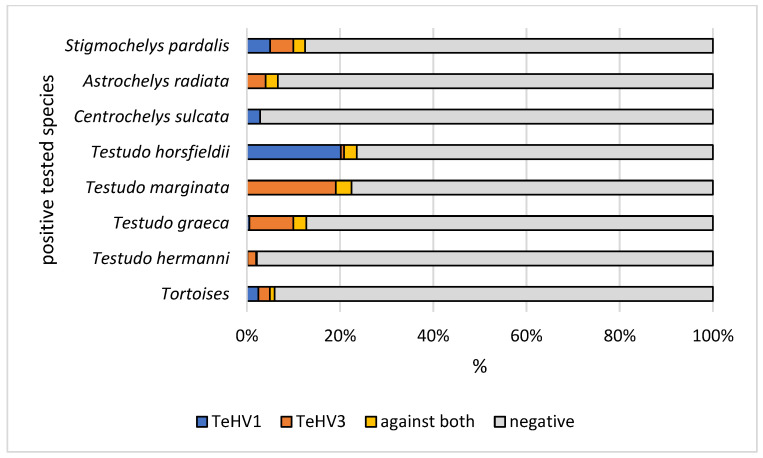
Herpesvirus (TeHV1 and TeHV3) antibody detection rates (titer 2 or higher) of the positive tested species (Hermann’s tortoises, *Testudo hermanni*; spur-thighed tortoises, *Testudo graeca*; marginated tortoises, *Testudo marginata*; Russian tortoises, *Testudo horsfieldii*; African spurred tortoise, *Centrochelys sulcata*; radiated tortoise, *Astrochelys radiata*; and leopard tortoise, *Stigmochelys pardalis*).

**Figure 2:**
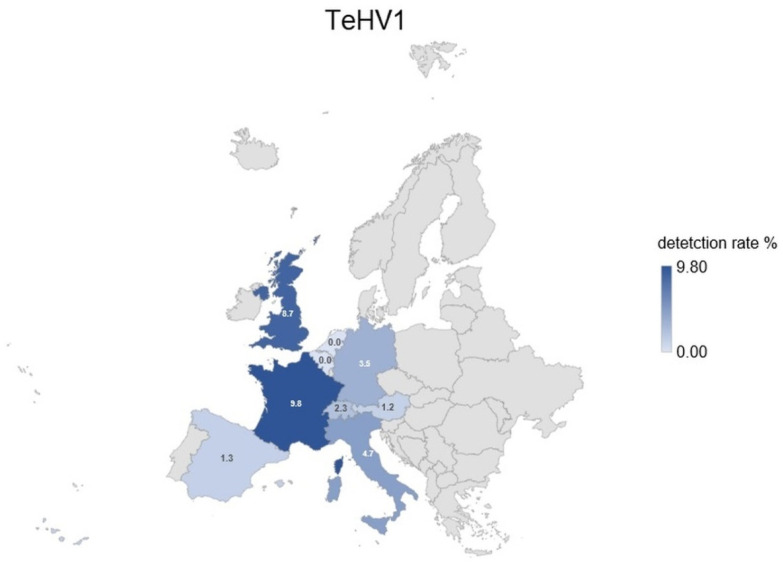
Herpesvirus (TeHV1) antibody detection rates (titer 2 or higher) divided into the countries of origin with more than 20 tested samples. Countries with lower numbers of samples submitted are shown in grey.

**Figure 3:**
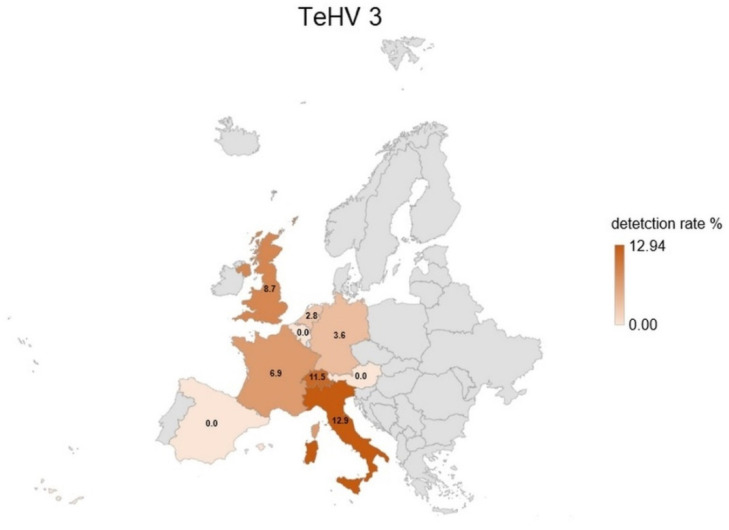
Herpesvirus (TeHV3) antibody detection rates (titer 2 or higher) divided into the countries of origin with more than 20 tested samples. Countries with lower numbers of samples submitted are shown in grey.

A total of 1728 samples from tortoises were evaluated. Antibodies (titer 2 or higher) against TeHV1 only were detected in 41 samples (2.37%; 95% CI 1.75–3.20%), and against TeHV3 only in 62 samples (3.59%; 95% CI 2.81–4.58%). Antibodies against both viruses were detected in samples from 19 animals (1.1%; 95% CI 0.71–1.71%), including one Hermann’s tortoise, five spur-thighed tortoises, three marginated tortoises (*Testudo marginata*), four Russian tortoises, two radiated tortoises (*Astrochelys radiata*), one leopard tortoise (*Stigmochelys pardalis*), and three tortoises for which the species was not provided. A total of 122 (7.06%; 95% CI 5.95–8.37%) of the animals tested had detectable antibodies against at least one of the herpesviruses used. The antibody detection rates differed significantly (*p* < 0.0001) for TeHV1 and TeHV3 (Table 1) depending on the tortoise species (Figure 1). There were significant differences (TeHV1 *p* = 0.0402; TeHV3 *p* = 0.0482) between years of sampling (Table 2). For TeHV1, antibody detection rates differed significantly (*p* = 0.0384) by season. The highest detection rate was in summer (5.59%; 95% CI 4.10-7.58%), and the lowest was in fall (1.25%; CI 0.53–2.87%) (Table 3). The detection rates for antibodies against TeHV3 did not differ significantly (*p* = 0.2617) by season (Table 3). TeHV1 antibody detection rates did not differ significantly (*p* = 0.7805) (Figure 2, Supplementary Table S1) by country. In contrast, TeHV3 antibody detection rates did differ significantly by country (*p* = 0.0090): no antibodies were detected in samples from Spain, Austria, Belgium, Luxemburg, Czech Republic, Poland, or Norway; and the highest detection rate, 12.94% (95% CI 7.38–21.70%), was found in samples from Italy (Figure 3, Supplementary Table S1).

**Figure 4:**
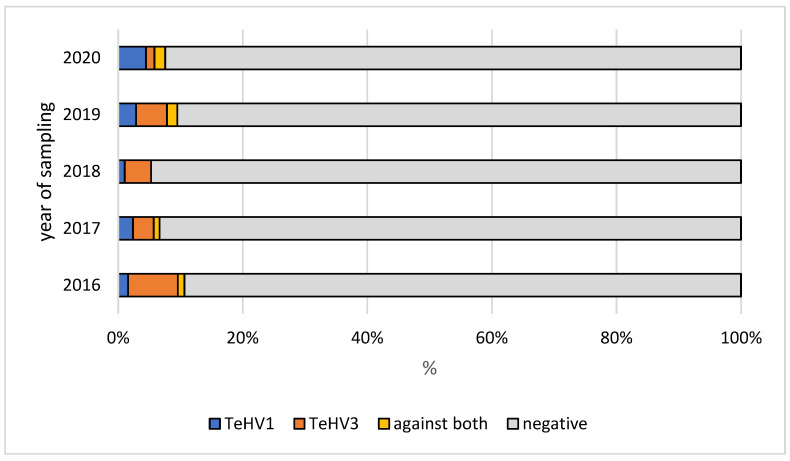
Herpesvirus (TeHV1 and TeHV3) antibody detection rates (titer 2 or higher) in five *Testudo* species (Hermann’s tortoises, *Testudo hermanni*; spur-thighed tortoises, *Testudo graeca*; marginated tortoises, *Testudo marginata*; Russian tortoises, *Testudo horsfieldii*; and Egyptian tortoises, *Testudo kleinmanni*) for the different years of sampling.

**Figure 5:**
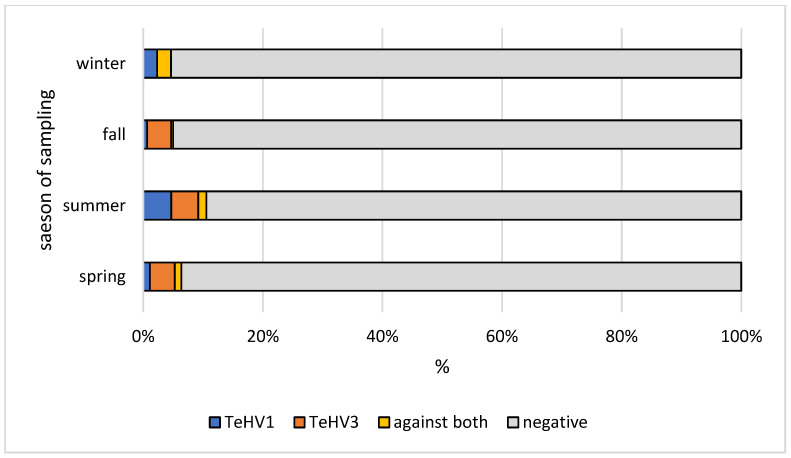
Herpesvirus (TeHV1 and TeHV3) antibody detection rates (titer 2 or higher) in five *Testudo* species (Hermann’s tortoises, *Testudo hermanni*; spur-thighed tortoises, *Testudo graeca*; marginated tortoises, *Testudo marginata*; Russian tortoises, *Testudo horsfieldii*; and Egyptian tortoises, *Testudo kleinmanni*) for the different seasons of sampling.

**Figure 6:**
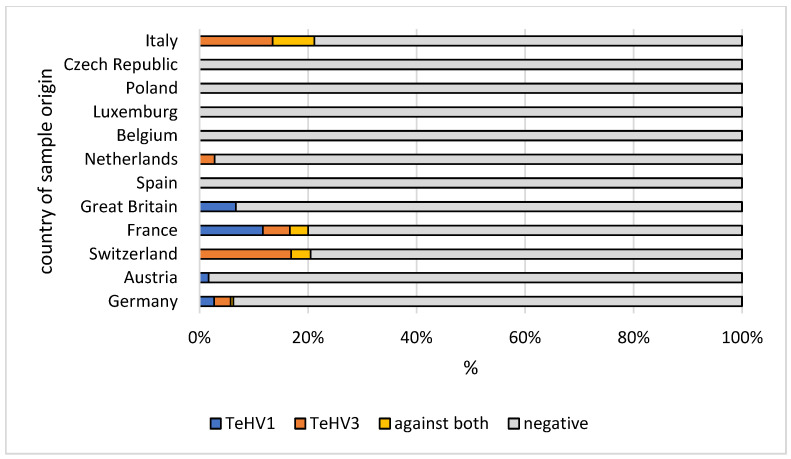
Herpesvirus (TeHV1 and TeHV3) antibody detection rates (titer 2 or higher) in five *Testudo* species (Hermann’s tortoises, *Testudo hermanni*; spur-thighed tortoises, *Testudo graeca*; marginated tortoises, *Testudo marginata*; Russian tortoises, *Testudo horsfieldii*; and Egyptian tortoises, *Testudo kleinmanni*) for the different countries of origin.

Evaluation of the results from samples from the five *Testudo* species (n = 1215) also showed some differences. There was a significant difference in antibody detection rates between the individual *Testudo* species for both virus types (*p* < 0.0001) (Table 1; Figure 1). No significant difference was found between the years of sampling for TeHV1 (*p* = 0.5328), but the differences were significant for TeHV3 (*p* = 0.0377) (Figure 4). Detection rates were found to differ significantly between seasons for TeHV1 (*p* = 0.0425) but not for TeHV3 (*p* = 0.6922) (Figure 5). For this subset of animals, as for all of the tortoises combined, country of sample origin did not significantly impact antibody detection rate for TeHV1 (*p* = 0.4751), whereas significant (*p* < 0.0001) differences were detected in detection rates for antibodies against TeHV3 based on country. The highest positivity rate (21.15%; 95% CI 12.24–34.03%) was found in Italy (Figure 6).

## 4. Discussion

Serology is an important tool in the diagnosis of herpesvirus infections in tortoises. It is also an important part of quarantine examinations in these animals. Since infected animals remain carriers, recognition of previous infection is a key to keeping infection out of a naïve group. A number of instances of herpesvirus introductions into previously negative collections followed by severe disease outbreaks have been documented [5,10,18], and both virus detection and serology have been used to detect herpesvirus infection in clinically healthy tortoises [5,18,19].

The antibody detection rate found here is similar to that found in a study reporting on PCR-based herpesvirus detection in chelonians in Europe over the same period [4]. In that study, an overall positivity rate of 6.22% was reported for chelonians in the family Testudinidae [4]. The vast majority of the herpesviruses detected in tortoises in that study were either TeHV1 (45.37%) or TeHV3 (53.24%). Studies comparing both virus detection and serology in the same animals have often shown higher virus detection rates than antibody detection rates [10,18,19]. In those cases in which samples were collected during an outbreak [10], it is possible that some animals had not yet developed a measurable immune response following infection. Both virus shedding and antibody detection have been reported in clinically healthy tortoises with no history of a recent disease outbreak [19]. In a study in which tortoises were repeatedly tested for the presence of antibodies over time, it was shown that animals that tested positive could also sporadically test negative [12]. In the present study, only serological testing, not virus detection, was carried out, so that direct comparison of the two could not be performed.

Host species has been shown to be a significant factor in infection rate for both TeHV1 and 3. TeHV1 has most often been detected in Russian tortoises, and this species is hypothesized to be the original host of this virus, although numerous other tortoise species have also been shown to be susceptible to infection [2,3,4,20], and infection has been associated with disease in several of these [2,20]. TeHV3 has been detected in a wide range of tortoise species [4]. It has been hypothesized that this virus may have co-evolved in spur-thighed tortoises [21], and this species and the closely related marginated tortoises have been reported to be more resistant to disease than some other species, e.g., Hermann’s tortoises, in outbreaks in mixed collections [10,18]. These host specificities are reflected in the antibody detection rates reported here. The highest rate of antibodies against TeHV1 was found in Russian tortoises. Distribution of the titers also showed a higher percentage of animals with titers > 16 in this species (Table 1, Figure 1). For TeHV3, the highest percentage of antibody positive animals were found for spur-thighed and marginated tortoises (Table 1, Figure 1). Previously published PCR-based virus detection rates in different animals in Europe over the same time period [4] revealed positivity rates that are comparable to the antibody detection rates found in this study. However, there were significant differences in PCR-based virus detection and antibody detection rates in Hermann’s tortoises between the two studies. A total of 4.66% of the samples from Hermann’s tortoises tested by PCR for herpesviruses (50 of 1072) were virus positive, the vast majority of which were TeHV3 [4]. In contrast, in the present study, antibodies against one of the viruses used were found in a significantly lower proportion of animals (2.25%, 18 of 797) (*p* = 0.0059), with extremely low titers of 2 or 4 in the majority of cases (12 of 18) (Table 1). While this is based on a different set of animals, so that direct comparison is not possible, it does support previous observations indicating that Hermann’s tortoises may be less able to mount a strong antibody response to infection with TeHV3 than, e.g., spur-thighed and marginated tortoises. This may help explain the apparent increased pathogenicity of these viruses in this species, although the immune response to herpesvirus infection in tortoises is not yet well understood and requires further study.

In several of the animals included in this study, antibodies were detected against both of the viruses used. Dual infection with TeHV1 and TeHV3 has been reported previously [3]. A study using serology for the detection of antibodies against a range of viruses in wild-caught spur-thighed tortoises in Turkey indicated that TeHV1 and 3 were co-circulating in that group [11]. Although TeHV1 and 3 have not been found to cross react serologically, it is possible that some of the antibodies detected were specific for other, similar antigens. It is important to note that virus neutralization testing with TeHV1 and 3 has not been validated for all of the species included in the present study and that some of the animals included could have been previously exposed to other herpesviruses not included in this study, with unknown cross reactivity to TeHV1 and 3. However, the use of a virus neutralization test indicates that the antibodies detected would have a functional use in the tortoises. The actual effect of neutralizing antibodies on disease development in herpesvirus infected tortoises has not, however, been sufficiently studied. In the present study, no information was provided on the clinical status of the animals tested.

Comparison of other factors that might influence virus and antibody detection rates between this study and the previous study on PCR-based virus detection [4] showed several other differences as well. Both PCR-based virus detection and antibody detection rates varied significantly depending on the year, but the years in which detection was highest for the two viruses differed between the two studies. Antibody detection rates may reflect a longer-term prevalence of these viruses in pet tortoises in Europe, and the results of the present study indicate that the immune response is subject to the same variations as virus detection by PCR. However, it is important to note that the samples used in both of these studies were submitted by veterinary practices from animals with unknown clinical status and histories, so that neither can fully reflect the true prevalence of herpesvirus infection in these animals.

Seasonality was also shown to influence PCR-based virus detection rates. The highest detection rates were found in the spring for both viruses [4]. In contrast, antibodies against both virus types were most often found in samples submitted in the summer. Since the immune system and antibody production in tortoises are dependent on environmental factors, this may reflect the reactivity of the immune system in summer rather than virus activity.

Both PCR-based virus detection and anti-TeHV3 antibody detection were significantly influenced by the country from which the samples were submitted. In both cases, the highest percentage of positive samples was found in samples from Italy, with most of these being TeHV3 and anti-TeHV3 antibodies [4]. Reasons for the high TeHV3 detection rate in Italy are not known. In the case of antibodies, it is possible that environmental factors could play a role in support of immunological reaction to infection, as the majority of the tortoises tested (Hermann’s, spur-thighed, and marginated tortoises) can be found in the wild there.

The reason for testing was not known for the animals included in this study, limiting the interpretation of results. It is likely that the animals sampled were biased toward those with a history of disease, since most were submitted by veterinarians, although submission for quarantine examinations and health checks likely also occurred in many cases.

Future studies exploring true prevalence and dynamics of herpesvirus infection in pet tortoises in Europe would be useful. This is, however, likely to be very difficult to organize, making data from diagnostic samples, as presented here, worth reporting. In addition, it would be helpful to continue studies of the herpesviruses that can infect tortoises. It is likely that not all herpesviruses capable of infecting this group of animals have been discovered yet. Finally, the differences in antibody detection rates between individual species and the possible implications for interactions between host species and specific viruses deserve further study. This should include studies elucidating the immune response to infection in various tortoise species and viral factors that are capable of influencing these reactions.

## 5. Conclusions

Antibody detection is an important tool in preventing the spread of herpesvirus infections in tortoises. Immune response to infection may, however, depend on several factors including virus type, host species, and environmental factors.

## Figures and Tables

**Table 1:** Detection rates of antibodies against testudinid herpesvirus 1 (TeHV1) and testudinid herpesvirus 3 (TeHV3) depending on species tested.

Species	Total Tested		TeHV1 Antibodies				TeHV3 Antibodies			
			Titer < 2	Titer 2 to 4	Titer 8	Titer ≥ 16	Titer < 2	Titer 2 to 4	Titer 8	Titer ≥ 16
Tortoise, species unknown	285	n	275	3	3	4	275	1	5	4
		%	96.50	1.05	1.05	1.40	96.50	0.35	1.75	1.40
		CI	93.66–98.08	0.36–3.04	0.36–3.04	0.55–3.55	93.66–98.08	0.06–1.96	0.75–4.03	0.55–3.55
*Testudo hermanni*	797	n	795	2	0	0	781	10	2	4
		%	99.75	0.25	0	0	98.00	1.25	0.25	0.50
		CI	99.09–99.93	0.07–0.91	0–0.48	0–0.48	96.76–98.76	0.68–2.29	0.07–0.91	0.19–1.28
*Testudo graeca*	180	n	174	3	1	2	158	9	3	10
		%	96.66	1.67	0.56	1.11	87.77	5.00	1.67	5.56
		CI	92.92–98.47	0.57–4.79	0.10–3.09	0.30–3.96	82.19–91.79	2.65–9.23	0.57–4.79	3.05–9.93
*Testudo marginata*	89	n	86	3	0	0	69	11	1	8
		%	96.63	3.37	0	0	77.53	12.36	1.12	8.99
		CI	90.55–98.85	1.15–9.45	0–4.14	0–4.14	67.82–84.96	7.04–20.79	0.02–6.09	4.63–16.75
*Testudo horsfieldii*	144	n	111	11	6	16	139	3	1	1
		%	77.08	7.64	4.17	11.11	96.54	2.08	0.69	0.69
		CI	69.56–8.19	4.32–13.16	1.93–8.80	6.96–17.29	92.13–98.51	0.71–5.94	0.12–3.82	0.12–3.82
*Testudo kleinmanni*	5	n	5	0	0	0	5	0	0	0
		%	100	0	0	0	100	0	0	0
		CI	56.55–100	0–43.45	0–43.45	0–43.45	56.55–100	0–43.45	0–43.45	0–43.45
*Centrochelys sulcata*	35	n	34	1	0	0	35	0	0	0
		%	97.14	2.86	0	0	100	0	0	0
		CI	85.46–99.49	0.51–14.54	0–9.89	0–9.89	90.11–100	0–9.89	0–9.89	0–9.89
*Astrochelys radiata*	75	n	73	1	0	1	70	3	1	1
		%	97.34	1.33	0	1.33	93.34	4.00	1.33	1.33
		CI	90.78–99.26	0.23–7.17	0–4.87	0.23–7.17	85.32–97.12	1.37–11.11	0.23–7.17	0.23–7.17
*Stigmochelys pardalis*	40	n	37	0	1	2	37	0	1	2
		%	92.50	0	2.50	5.00	92.50	0	2.50	5.00
		CI	80.14–97.42	0–8.76	0.44–12.88	1.38–16.50	80.14–97.42	0–8.76	0.44–12.88	1.38–16.50
*Aldabrachelys gigantea*	12	n	12	0	0	0	12	0	0	0
		%	100	0	0	0	100	0	0	0
		CI	75.75–100	0–24.25	0–24.25	0–24.25	75.75–100	0–24.25	0–24.25	0–24.25
*Chelonoidis nigra*	2	n	2	0	0	0	2	0	0	0
		%	100	0	0	0	100	0	0	0
		CI	34.24–100	0–65.76	0–65.76	0–65.76	34.24–100	0–65.76	0–65.76	0–65.76
*Chelonoidis carbonarius*	14	n	14	0	0	0	14	0	0	0
		%	100	0	0	0	100	0	0	0
		CI	78.47–100	0–21.53	0–21.53	0–21.53	78.47–100	0–21.53	0–21.53	0–21.53
*Chelonoidis denticulatus*	3	n	3	0	0	0	3	0	0	0
		%	100	0	0	0	100	0	0	0
		CI	43.85–100	0–56.15	0–56.15	0–56.15	43.85–100	0–56.15	0–56.15	0–56.15
*Geochelone elegans*	9	n	9	0	0	0	9	0	0	0
		%	100	0	0	0	100	0	0	0
		CI	70.09–100	0–29.91	0–29.91	0–29.91	70.09–100	0–29.91	0–29.91	0–29.91
*Geochelone platynota*	7	n	7	0	0	0	7	0	0	0
		%	100	0	0	0	100	0	0	0
		CI	64.57–100	0–35.43	0–35.43	0–35.43	64.57–100	0–35.43	0–35.43	0–35.43
*Astrochelys yniphora*	5	n	5	0	0	0	5	0	0	0
		%	100	0	0	0	100	0	0	0
		CI	56.55–100	0–43.45	0–43.45	0–43.45	56.55–100	0–43.45	0–43.45	0–43.45
*Kinixys* sp.	3	n	3	0	0	0	3	0	0	0
		%	100	0	0	0	100	0	0	0
		CI	43.85–100	0–56.15	0–56.15	0–56.15	43.85–100	0–56.15	0–56.15	0–56.15
*Indotestudo elongata*	4	n	4	0	0	0	4	0	0	0
		%	100	0	0	0	100	0	0	0
		CI	51.01–100	0–48.99	0–48.99	0–48.99	51.01–100	0–48.99	0–48.99	0–48.99
*Gopherus berlandieri*	1	n	1	0	0	0	1	0	0	0
		%	100	0	0	0	100	0	0	0
		CI	20.65–100	0–79.35	0–79.35	0–79.35	20.65–100	0–79.35	0–79.35	0–79.35
*Malacochersus tornieri*	6	n	6	0	0	0	6	0	0	0
		%	100	0	0	0	100	0	0	0
		CI	60.97–100	0–39.03	0–39.03	0–39.03	60.97–100	0–39.03	0–39.03	0–39.03
*Homopus* sp.	1	n	1	0	0	0	1	0	0	0
		%	100	0	0	0	100	0	0	0
		CI	20.65–100	0–79.35	0–79.35	0–79.35	20.65–100	0–79.35	0–79.35	0–79.35
*Manouria* sp.	1	n	1	0	0	0	1	0	0	0
		%	100	0	0	0	100	0	0	0
		CI	20.65–100	0–79.35	0–79.35	0–79.35	20.65–100	0–79.35	0–79.35	0–79.35
*Pyxis* sp.	6	n	6	0	0	0	6	0	0	0
		%	100	0	0	0	100	0	0	0
		CI	60.97–100	0–39.03	0–39.03	0–39.03	60.97–100	0–39.03	0–39.03	0–39.03
*Chersina angulata*	2	n	2	0	0	0	2	0	0	0
		%	100	0	0	0	100	0	0	0
		CI	34.24–100	0–65.76	0–65.76	0–65.76	34.24–100	0–65.76	0–65.76	0–65.76
*Psammobates* sp.	2	n	2	0	0	0	2	0	0	0
		%	100	0	0	0	100	0	0	0
		CI	34.24–100	0–65.76	0–65.76	0–65.76	34.24–100	0–65.76	0–65.76	0–65.76
**Total**	1728	n	1668	24	11	25	1647	37	14	30
		%	96.53	1.39	0.64	1.45	95.31	2.14	0.81	1.74
		CI	95.56–97.29	0.94–2.06	0.36–1.14	0.98–2.13	94.21–96.21	1.56–2.94	0.48–1.36	1.22–2.47

n: number in each category; CI: 95% confidence interval.

**Table 2:** Detection rates of antibodies against testudinid herpesvirus 1 (TeHV1) and testudinid herpesvirus 3 (TeHV3) depending on year of sampling. Results shown as: number (n), percent (%), 95% confidence interval (CI).

Year	Total Tested		TeHV1 Antibodies				TeHV3 Antibodies			
			Titer < 2	Titer 2 to 4	Titer 8	Titer ≥ 16	Titer < 2	Titer 2 to 4	Titer 8	Titer ≥ 16
2016	305	n	299	2	1	3	285	5	5	10
		%	98.03	0.66	0.33	0.98	93.44	1.64	1.64	3.28
		CI	95.77–99.09	0.18–2.37	0.06–1.84	0.33–2.85	90.09–95.71	0.70–3.78	0.70–3.78	1.79–5.93
2017	303	n	292	4	3	4	290	6	2	5
		%	96.37	1.32	0.99	1.32	95.71	1.98	0.66	1.65
		CI	93.62–97.96	0.51–3.34	0.34–2.87	0.51–3.34	92.80–97.48	0.91–4.25	0.18–2.70	0.71–3.80
2018	381	n	377	1	1	2	369	6	0	6
		%	98.96	0.26	0.26	0.52	96.86	1.57	0.0	1.57
		CI	97.33–99.59	0.05–1.47	0.05–1.47	0.14–1.89	94.58–98.19	0.72–3.39	0–1.00	0.72–3.39
2019	350	n	331	9	4	6	326	13	5	6
		%	94.58	2.57	1.14	1.71	93.15	3.71	1.43	1.71
		CI	91.68–96.50	1.36–4.81	0.44–2.90	0.79–3.68	90.00–95.35	2.18–6.24	0.61–3.30	0.79–3.68
2020	389	n	369	8	2	10	377	7	2	3
		%	94.86	2.06	0.51	2.57	96.92	1.80	0.51	0.77
		CI	92.19–96.65	1.05–4.01	0.14–1.85	1.40–4.67	94.69–98.23	0.87–3.67	0.14–1.85	0.26–2.24
**Total**	1728		1668	24	11	25	1647	37	14	30

**Table 3:** Detection rates of antibodies against testudinid herpesvirus 1 (TeHV1) and testudinid herpesvirus 3 (TeHV3) depending on season of sampling. Results shown as: number, percent, 95% confidence interval (CI).

Season	Total Tested		TeHV1 Antibodies				TeHV3 Antibodies			
			Titer < 2	Titer 2 to 4	Titer 8	Titer ≥ 16	Titer < 2	Titer 2 to 4	Titer 8	Titer ≥ 16
Spring	529	n	515	5	5	4	505	9	6	9
		%	97.34	0.95	0.95	0.76	95.47	1.70	1.13	1.70
		CI	95.60–98.41	0.41–2.20	0.41–2.20	0.30–1.93	93.33–96.93	0.90–3.20	0.52–2.45	0.90–3.20
Summer	680	n	642	17	6	15	642	17	5	16
		%	94.41	2.50	0.88	2.21	94.41	2.50	0.74	2.35
		CI	92.42–95.90	1.57–3.97	0.40–1.91	1.34–3.61	92.42–95. 90	1.57–3.97	0.32–1.72	1.45–3.78
Fall	402	n	397	2	0	3	384	11	3	4
		%	98.75	0.50	0	0.75	95.51	2.74	0.75	1.00
		CI	97.13–99.47	0.14–1.80	0–0.95	0.26–2.18	93.03–97.15	1.54–4.84	0.26–2.18	0.39–2.54
Winter	117	n	114	0	0	3	116	0	0	1
		%	97.44	0	0	2.56	99.15	0	0	0.85
		CI	92.74–99.13	0–3.18	0–3.18	0.87–7.26	95.32–99.85	0–3.18	0–3.18	0.15–4.68
**Total**	1728		1668	24	11	25	1647	37	14	30

## Data Availability

The data that support the findings of this study are available from the corresponding author upon reasonable request.

## Supplementary Materials

The following supporting information can be downloaded at: https://www.mdpi.com/article/10.3390/ani12172298/s1. Table S1: Detection rates of antibodies against testudinid herpesvirus 1 (TeHV1) and testudinid herpesvirus 3 (TeHV3) depending on country of origin. Results shown as: number, percent, 95% confidence interval (CI).
