# Supplementary material for: Antibodies against Two Testudinid Herpesviruses in Pet Tortoises in Europe
Source: Animals (Basel). 2022 Sep 5;12(17):2298. doi: 10.3390/ani12172298 (PMC9454543; doi:10.3390/ani12172298)
Supplement: Supplementary file 1 [file animals-12-02298-s001.zip › animals-1861180-supplementary.pdf]

Supplementary Table S1 Detection rate of antibodies against testudinid herpesvirus 1 (TeHV1) and testudinid herpesvirus 3 (TeHV3) depending on country of sample origin. Results shown as: number (n), percent, 95% confidence interval (CI).

Country	Total tested		TeHV1 antibodies				TeHV3 antibodies			
			Titer <2	Titer 2 to 4	Titer 8	Titer ≥16	Titer <2	Titer 2 to 4	Titer 8	Titer ≥16
Germany	1102	n	1064	14	8	16	1062	17	10	13
		%	96.55	1.27	0.73	1.45	96.37	1.54	0.91	1.18
		CI	95.33-97.49	0.75-2.11	0.36-1.42	0.90-2.34	95.09-97.32	0.96-2.45	0.5-1.67	0.69-2.01
Switzerland	174	n	170	2	1	1	154	7	3	10
		%	97.71	1.15	0.57	0.57	88.51	4.02	1.72	5.75
		CI	94.24-99.10	0.32-4.09	0.10-3.18	0.10-3.18	82.92-92.44	1.96-8.07	0.59-4.94	3.15-10.26
France	102	n	92	3	1	6	95	5	0	2
		%	90.20	2.94	0.98	5.88	93.14	4.90	0.0	1.96
		CI	82.89-94.59	1.00-8.29	0.17-5.35	2.72-12.24	86.51-96.64	2.11-10.96	0-3.63	0.5485-10.55
Italy	85	n	81	3	0	1	74	7	0	4
		%	95.29	3.53	0.0	1.18	87.05	8.24	0.0	4.71
		CI	88.51-98.15	1.21-9.87	0-4.32	0.21-6.37	78.30-92.62	4.05-16.04	0-4.32	1.85-11.49

Austria	81	n	80 (%; CI %)	0 (%; CI %)	0 (%; CI %)	1 (%; CI %)	81 (%; CI %)	0 (%; CI %)	0 (%; CI %)	0 (%; CI %)
		%	98.77	0.0	0.0	1.23	100	0.0	0.0	0.0
		CI	93.34-99.78	0-4.53	0-4.53	0.22-6.66	95.47-100	0-4.53	0-4.53	0-4.53
Spain	75	n	74	1	0	0	75	0	0	0
		%	98.67	1.33	0.0	0.0	100	0. 0	0. 0	0. 0
		CI	92.83-99.77	0.23-7.17	0-4.87	0-4.87	95.13-100	0-4.87	0-4.87	0-4.87
Netherlands	36	n	36	0	0	0	35	1	0	0
		%	100	0.0	0.0	0.0	97.22	2.78	0.0	0.0
		CI	90.36-100	0-9.64	0-9.64	0-9.64	85.83-99.51	0.49-14.17	0-9.64	0-9.64
Belgium	29	n	29	0	0	0	29	0	0	0
		%	100	0.0	0.0	0.0	100	0.0	0.0	0.0
		CI	88.30-100	0-11.70	0-11.70	0-11.70	88.30-100	0-11.70	0-11.70	0-11.70
United Kingdom	23	n	21	1	1	0	21	0	1	1
		%	91.30	4.35	4.35	0.0	91.30	0.0	4.35	4.35
		CI	67.88-95.46	0.77-20.99	0.77-20.99	0-14.31	73.20-97.58	0-14.31	0.77-20.99	0.77-20.99
Luxemburg	7	n	7	0	0	0	7	0	0	0

		%	100	0.0	0.0	0.0	100	0.0	0.0	0.0
		CI	64.57-100	0-35.43	0-35.43	0-35.43	64.57-100	0-35.43	0-35.43	0-35.43
Czech Republic	5	n	5	0	0	0	5	0	0	0
		%	100	0.0	0.0	0.0	100	0.0	0.0	0.0
		CI	56.55-100	0-43.45	0-43.45	0-43.45	56.55-100	0-43.45	0-43.45	0-43.45
Poland	5	n	5	0	0	0	5	0	0	0
		%	100	0.0	0.0	0.0	100	0.0	0.0	0.0
		CI	56.55-100	0-43.45	0-43.45	0-43.45	56.55-100	0-43.45	0-43.45	0-43.45
Norway	4	n	4	0	0	0	4	0	0	0
		%	100	0.0	0.0	0.0	100	0.0	0.0	0.0
		CI	51.01-100	0-48.99	0-48.99	0-48.99	51.01-100	0-48.99	0-48.99	0-48.99
Total	1728		1668	24	11	25	1647	37	14	30
